# *MIMIC-III-Ext-PPG*, a PPG-based Benchmark Dataset for Cardiovascular and Respiratory Signal Analysis

**DOI:** 10.1038/s41597-026-07335-8

**Published:** 2026-04-28

**Authors:** Mohammad Moulaeifard, Marie Kutscher, Philip J. Aston, Peter H. Charlton, Nils Strodthoff

**Affiliations:** 1 AI4Health Department, Oldenburg University, Oldenburg, Germany; 2 Department of Data Science and AI, National Physical Laboratory, Teddington, United Kingdom; 3 School of Mathematics and Physics, University of Surrey, Guildford, United Kingdom; 4 Department of Public Health and Primary Care, University of Cambridge, Cambridge, United Kingdom

**Keywords:** Biomarkers, Health care, Biomarkers

## Abstract

We present *MIMIC-III-Ext-PPG*, a large-scale, quality-assessed photoplethysmography (PPG) dataset derived from the matched waveform subset of MIMIC-III. Our dataset provides 30-second PPG segments with annotations tailored for various cardiovascular and respiratory analyses. In particular, with 6.3 million segments from 6,189 subjects, it represents the largest publicly available resource for heart rhythm classification, with heart rhythm annotations derived from bedside charted observations. For subsets where arterial blood pressure (ABP), respiratory (RESP), and/or electrocardiography (ECG) signals are available, we also provide systolic/diastolic blood pressure, respiratory rate, and heart rate annotations, extracted using best practice from the underlying signals. We provide signal quality assessments for all signals. This ensures a high-quality, publicly available dataset of unprecedented size that can be used as a benchmarking resource for machine learning approaches for a broad range of prediction tasks, which remains easily extendable by leveraging additional clinical metadata from the MIMIC-III clinical database.

## Background & Summary

Physiological signals, such as photoplethysmography (PPG), electrocardiography (ECG), arterial blood pressure (ABP), and respiration (RESP) signals, are critical for monitoring cardiovascular and respiratory function in intensive care units (ICUs). These signals provide a detailed temporal view of physiological function and are widely used in clinical and signal processing research, for tasks such as heart rhythm classification, blood pressure estimation, respiratory analysis, and signal quality assessment. The focus of this dataset lies on the PPG signal, which is an optical signal acquired non-invasively via pulse oximeters in critical care, and is also widely measured by consumer wearables such as smartwatches. Prominent applications include arrhythmia detection and estimation of cuffless blood pressure (BP), heart rate (HR), and respiratory rate (RR).

Several PPG-based datasets have been proposed to train and validate machine learning algorithms to approach these downstream prediction tasks, see Table 1 for a comparison of large-scale PPG datasets. Existing datasets fall short in several respects. First, their size: Typical PPG datasets are mostly relatively small, either including relatively few subjects (e.g. Deepbeat), and/or having short signal durations (e.g. UK Biobank). *MIMIC-III-Ext-PPG*^1^ is the largest public dataset to-date for PPG-based heart rhythm classification. Second, their accompanying features: Many PPG datasets include at most gender information, and few include biometrics, ethnicity, and comorbidities (UK Biobank being a notable exception). Third, tasks: Most datasets can only be used to address a single task, and the only dataset that can be used for heart rhythm classification (DeepBeat) contains only two classes of heart rhythm. Fourth, availability: Not all datasets are publicly available. Fifth, quality: Not all datasets provide signal quality assessments for all samples and channels or carry out rigorous filtering to remove signals that are unsuitable for analysis.

**Table 1:** Comparison of large-scale PPG datasets.

Name	Detail	MIMIC-BP^22^	PulseDB^12^	DeepBeat^23^	Aurora-BP^24^	UK Biobank^25^	MIMIC-III-Ext-PPG
Source		MIMIC-III	MIMIC-III + VitalDB	cohorts	study	study	MIMIC-III
Sensor		fingertip	fingertip	wrist	wrist	fingertip	fingertip
Population		ICU	ICU/intra-op.	ambulatory	population	population	ICU
Size	Subjects	1,524	5,361	175	1,125	205,357	6,131^6^
	Total Duration (h)	380	14,570	1,075^3^	211	685	40,970^6^
	Segment Duration (s)	30	10	25	9.1-87.1	10-15	30
Metadata	Demographics	✗^2^	*✓*	✗	*✓*	*✓*	*✓*
	Ethnicity	✗^2^	✗^2^	✗	✗	*✓*	*✓*
	Biometrics	✗^2^	✗^2^	✗	*✓*	*✓*	*✓*
	Comorbidities	✗^2^	✗^2^	✗	*✓*	*✓*	*✓*
Tasks	Heart Rhythm	✗	✗	*✓*(2 classes)	✗^2^	✗^4^	*✓*(26 classes)
	BP	*✓*	*✓*	✗	*✓*	✗^4^	*✓*
	HR	✗	✗	✗	✗^2^	✗^4^	*✓*
	RR	✗	✗	✗	✗^2^	✗^4^	*✓*
Quality	SQI	✗	✗^7^	*✓*	*✓*^0^	✗^4^	*✓*
Availability	Open data	*✓*	*✓*	*✓*	✗^1^	✗^5^	*✓*

**Legend**: *✓* = available, **✗**= unavailable, BP: blood pressure, HR: heart rate, RR: respiratory rate, SQI: availability of signal quality index, ^0^not distributed as part of the dataset but provided by third party publications^26^, ^1^Sample data is open; full dataset requires access request, ^2^can be retrieved from underlying source datasets, ^3^only non-overlapping segments, ^4^only including targets measured simultaneously with the PPG-signal, ^5^access request and access fee required, ^6^statistics for the heart rhythm case, see Table 2 for other tasks, ^7^the low quality segments were excluded according to the procedure described in^12^; however, no explicit SQI was defined.

To address these limitations, we introduce *MIMIC-III-Ext-PPG*, a large-scale, rigorously curated, quality-assessed, and multimodal benchmark dataset derived from the matched subset of the MIMIC-III Waveform Database^2^. This subset combines physiological signals and electronic health record data from both the CareVue and MetaVision systems, harmonized through consistent preprocessing.

It should be noted that in our work, the term “record” refers to a single WFDB file. We identified recordings containing PPG (denoted PLETH in MIMIC) signals from over 10,282 ICU subject admissions and paired them with corresponding features from the electronic health record (known as clinical chart events in MIMIC), including cardiac heart rhythm annotations (where the term heart rhythm refers to the heart rhythm category in the chart events, which includes both cardiac heart rhythms as well as conduction disturbances), demographic information, and ICD-9 discharge codes^3^, which were systematically converted to ICD-10 codes^4^ for interoperability across systems. It is worth stressing that *MIMIC-III-Ext-PPG* is not only the largest PPG-dataset for heart rhythm prediction but also the most fine-grained, covering 26 different heart rhythm types.

**Fig. 1:**
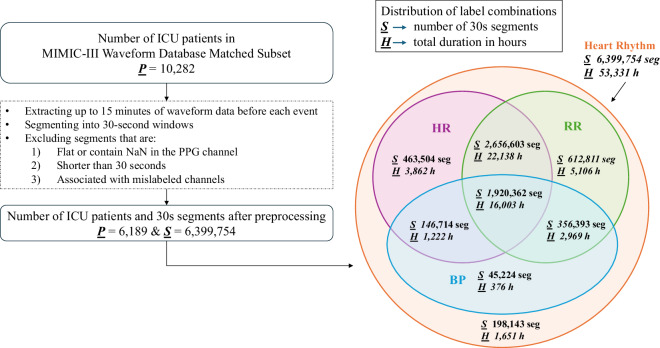
Patient selection diagram and Venn diagram of dataset subsets stratified according to available annotations.

Where available, additional synchronized physiological signals are also provided alongside PPG signals, consisting of ECG (lead II), ABP, and RESP signals. We extracted up to 15 minutes of signals preceding each annotated heart rhythm event, segmented into 30-second segments for analysis of heart rhythm, RR, BP, and HR. Figure 1 shows both the patient selection process and the distribution of label combinations used in the study.

All waveform data were provided in WFDB format, with cross-referenced annotations in synchronized metadata tables to provide alignment between signal and clinical domains. To facilitate high-quality downstream analysis, signal quality indices (SQIs) were calculated on a per-signal modality basis. These comprised correlation-based approaches, consistency of beat detection, and template-matching, derived from state-of-the-art techniques in the literature^5,6^. We summarize the basic characteristics of *MIMIC-III-Ext-PPG* stratified according to different prediction tasks in Table 2 and Fig. 1.

**Table 2:** Summary of *MIMIC-III-Ext-PPG* subsets for different prediction tasks.

Metric	Heart Rhythm	RR	HR	BP
**Number of subjects**	6,189	4,695	5,924	2,391
**Number of segments (30s)**	6,399,754	5,546,169	5,187,183	2,468,693
**Total Duration (h)**	~53,331	~46,218	~43,227	~20,572
**Age (years, mean ± SD)**	64.1 ± 17.0	65.0 ± 16.1	63.9 ± 16.9	63.2 ± 16.2
**Weight (kg, mean ± SD)**	82.2 ± 22.6	82.0 ± 21.8	82.3 ± 22.6	83.2 ± 21.9
**Height (cm, mean ± SD)**	169.5 ± 10.5	169.7 ± 10.5	169.4 ± 10.5	169.7 ± 10.4
**Gender (female, %)**	43.9	43.3	44.0	42.2
**Ethnicity (%)**				
**- White**	72.0	73.5	72.0	71.4
**- Black**	9.2	9.3	9.1	7.7
**- Hispanic**	4.1	3.8	4.1	3.8
**- Asian**	2.8	2.8	2.9	3.0
**- Other**	11.9	10.6	11.9	14.1

All of the age, weight, height, and ethnicity values are based on the initial visits of the unique subjects.

## Methods

### Data Preparation

To construct the *MIMIC-III-Ext-PPG* dataset, we implemented a systematic pipeline to extract and harmonize physiological waveforms and associated clinical metadata from the MIMIC-III matched waveform and clinical subsets. The preparation involved the following steps:

1. **Waveform Selection:** All waveform records from the MIMIC-III Waveform Database Matched Subset were investigated for available channels. For this study, we selected only waveform records that contained a PPG signal (denoted PLETH in MIMIC).
2. **Electronic Health Record Metadata Extraction:** 480,678 heart rhythm chart events from just those records with a PLETH signal, as well as demographic feature measurements, were obtained from the MIMIC-III clinical database^7^. Data were extracted via both the CareVue and MetaVision systems, depending on the time of patient admission.
3. **Event matching:** For each waveform identified in the previous step, we checked whether a heart rhythm chart event occurred within the waveform’s duration. If so, we extracted (i) the current heart rhythm annotation, and (ii) the immediately preceding heart rhythm event.
4. **Signal segment extraction:** For each matched heart rhythm event, we extracted a segment of the waveform ending at the chart time of the heart rhythm event. The segment started either from the timestamp of the preceding heart rhythm annotation or from a point at most 15 minutes prior, whichever occurred later. Then we split the segments into 30-second waveform segments (the remainder of the segment, which was less than 30 seconds in length, was deleted).
5. **Harmonizing heart rhythm annotations:** Given that the CareVue and MetaVision follow different annotation formats, we unified the heart rhythm labels across both systems into a consistent vocabulary, see acronyms summarized in Table 3.

**Table 3:** Heart rhythm events in the entire dataset stratified according to subjects and samples.

Code	Rhythm Name	Superclasses	unqiue subjects	30-second segments
SR	Sinus rhythm	Normal Sinus Rhythm	5,316	3,950,724
STACH	Sinus tachycardia	Sinus Node Disorders	2,838	1,192,025
AF	Atrial fibrillation	Atrial Arrhythmias	1,132	597,769
SBRAD	Sinus bradycardia	Sinus Node Disorders	1,608	198,432
VPACE	Ventricular pacing	Artificial Pacing	282	126,810
1AVB	1^st^ degree AV block	AV Conduction Blocks	304	111,729
AVPACE	Atrioventricular pacing	Artificial Pacing	166	67,719
APACE	Atrial pacing	Artificial Pacing	145	44,415
AFLT	Atrial flutter	Atrial Arrhythmias	210	44,811
LBBB	Left bundle branch block	Bundle Branch Blocks	45	22,667
RBBB	Right bundle branch block	Bundle Branch Blocks	43	13,092
JR	Junctional rhythm	Junctional Rhythms	78	6,823
SVTACH	Supraventricular tachycardia	Atrial Arrhythmias	193	6,342
SARRH	Sinus arrhythmia	Sinus Node Disorders	105	7,224
2AVBM1	2^nd^ degree AV block Mobitz type 1	AV Conduction Blocks	20	1,768
3AVB	3^rd^ degree AV block	AV Conduction Blocks	27	1,360
VTACH	Ventricular tachycardia	Ventricular Arrhythmias	53	1,273
MATACH	Multifocal atrial tachycardia	Atrial Arrhythmias	16	1,725
2AVBM2	2^nd^ degree AV block Mobitz type 2	AV Conduction Blocks	19	705
WAPACE	Wandering atrial pacemaker	Atrial Arrhythmias	7	854
JTACH	Junctional tachycardia	Junctional Rhythms	16	571
OTHER	Other	Unclassified	9	349
VFIB	Ventricular fibrillation	Ventricular Arrhythmias	9	147
IDIOV	Idioventricular rhythm	Ventricular Arrhythmias	7	111
ASYS	Asystole	Cardiac Arrest	15	113
PATACH	Paroxysmal atrial tachycardia	Atrial Arrhythmias	7	196
**Total**				**6,399,754**
6. **Discharge diagnoses:** Using hospital admission IDs (HADM_ID), we retrieved all ICD-9 discharge diagnoses and converted them to ICD-10 codes. These were then propagated along the ICD-10 hierarchy and truncated to the three-digit level. The cardiovascular codes were later used for patient stratification to define stratified folds.
7. **Demographics and biometrics:** Demographic and biometric variables, including patient age, sex, weight, height, and ethnicity, were extracted from structured fields in the MIMIC-III clinical database.
8. **Stratified splits:** To facilitate the benchmarking of machine learning algorithms on the dataset, we split the dataset into 10 non-overlapping folds based on unique patient identifiers. The stratification process^8,9^ aimed to balance heart rhythm events, age distributions (6 strata), and cardiovascular ICD-10 discharge diagnoses across folds.
9. **Signal Storage:** Extracted segments were saved in the WFDB format and included the PPG (PLETH) signal and, where available, ECG (lead II), ABP, and RESP signals.

### Incorporation of Supplementary Annotations and Signal Quality Indicators

Each 30-second segment was further augmented with supplementary annotations and signal quality assessments derived from the corresponding waveforms:

1. **ABP, PPG, and ECG** signals were examined in three sequential 10-second intervals (0-10s, 10-20s, and 20-30s), yielding a three-element vector for each.
2. **RESP** was evaluated across the entire 30-second duration.

The following section outlines the procedures used to generate these additional annotations and quality metrics.

#### Supplementary Annotation of Physiological Parameters

The dataset was further annotated with key physiological parameters derived from the available waveform data:

1. **HR from ECG (if available):** HR was derived from every beat-to-beat interval within each 10-second window with the QRS detection algorithm from NeuroKit (^10^), with HR values calculated from the median RR intervals (^6^). For each 30-second segment, a 3-element vector was extracted for both the median and the IQR and reported accordingly.
2. ** Systolic and diastolic blood pressure (SBP and DBP) from ABP (if available):** SBP and DBP were determined using the slope sum function (SSF) algorithm for beat detection (^11^) within each 10-second window. This process yielded the three-element vectors per 30-second segment (median and IQR per 10-second), along with aggregated metrics (median and IQR per 30s) across the full 30-second segment.
3. **RR from RESP (if available):** RR was computed over the full 30-second segment using the method described in^5^. Instantaneous RR values were obtained from each detected breath interval, and the final 30-second segment-level RR was summarized using the median and IQR.

#### Enrichment with Signal Quality Assessments

**Fig. 2:**
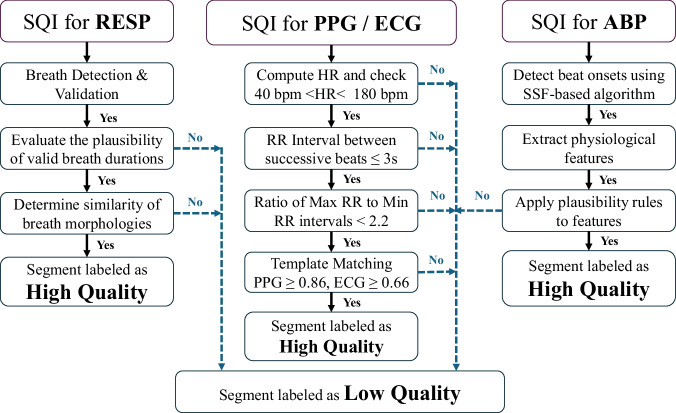
Flowchart summarizing the signal quality assessment pipeline for RESP based on^5^, PPG/ECG based on^6^, and ABP based on^11^. Each column illustrates the sequential steps and criteria used to evaluate signal quality for the corresponding physiological signal type.

We introduced a comprehensive signal quality evaluation pipeline specifically designed for multi-signal physiological inputs, such as RESP, PPG, ECG, and ABP signals (Fig. 2). Signal quality was quantified using an integer-based SQI. Supplementary Table S1 presents the SQI code map, summarizing the possible outcomes of the quality assessment applied in this study. By default, 30-second segments or 10-second windows that passed all high quality checks were labeled as *high quality* (SQI =  +1), and those failing one or more high quality checks while still usable, were considered *low quality* (SQI = 0). 30-second segments or 10-second windows with structural issues that prevented SQI computation were assigned specific negative SQI codes indicating the failure reason.

1. **General Assessment:** All signals were initially screened for flatlines and NaN values. A signal was labeled as *Flat* if it met either of two criteria proposed by^12^: (1) four or more consecutive samples equal to the minimum or maximum value, or (2) a single repeated value lasting more than one second. For RESP, the entire 30-second segment was labeled as *Flat* if constant for more than five seconds. Flat signals were assigned SQI =  −2, and those containing NaN values were assigned SQI =  −3.
2. **RESP SQI:** Following^5^, 30-second segments were evaluated for valid respiratory cycles, morphological consistency via template matching, and signal variability. SQI values of  −11,  −12, and  −13 were used for the cases with undetectable peaks or troughs, insufficient breath cycles, or inadequate segments for template matching, respectively.
3. **PPG and ECG SQI:** Following^6^, signal windows were assessed for heart rate plausibility, beat interval consistency, and morphology via template correlation. SQI values of  −14,  −15, and  −16 indicated missing RR intervals, RR 10-second windows that were too short, or insufficient segments for template matching. Additionally, 10-second windows with invalid or insufficient R-peaks (ECG) or PPG peaks were labeled  −17 and  −18, respectively.
4. **ABP SQI:** Based on the^11,13^, 10-second windows were assessed for beat onset detection, plausibility of SBP/DBP/HR features, and noise artifacts. A failure of beat onset detection was indicated by SQI =  −19.

It is worth noting that *MIMIC-III-Ext-PPG* includes only those segments whose PPG SQI vectors contain non-negative values (i.e., 0 or 1) across all three 10-second windows, indicating analyzable signals of either low or high quality.

## Data Records

The described *MIMIC-III-Ext-PPG* dataset comprises 30-second PPG segments, additional simultaneous signals, and comprehensive metadata, which makes it easy to develop and benchmark machine learning models on this dataset. The dataset is publicly accessible through PhysioNet^1^.

### Waveform data

All waveform signals (PPG, ECG, ABP, RESP) are sampled at 125 Hz and segmented into 30-second segments. The waveform data is organized in a folder structure based on subject IDs (see Supplementary Figure S1). At the top level, the data is categorized by the first two digits of the zero-padded subject ID. These folders then contain separate folders for each subject ID, which contain each patient’s signal files.

### Metadata

The metadata is provided as a flat csv-file “metadata.csv”, which contains all the available metadata for each of the 30-second segments. Whilst the metadata corresponding to RESP is reported per 30-second segment, the metadata for the other signals (ABP, ECG, and PPG) are presented for each 10-second window within the 30-second segment.

A comprehensive description of all dataset variables is provided in Supplementary Table S2.

### Descriptive summary

We summarize the different subsets of *MIMIC-III-Ext-PPG* in Table 2 in terms of demographics and biometrics. The covered heart rhythm event distribution is summarized in Table 3. It covers 26 unique heart rhythm statements that can be broadly categorized into 10 different superclasses, ranging from sinus rhythm to various arrhythmias and conduction disturbances. To the best of our knowledge, such a detailed and fine-grained annotation has not been provided for PPG-based datasets in the literature, enabling entirely new perspectives for PPG-based heart rhythm analysis.

## Technical Validation

In this section, we present the results of the technical validation along with the final data distribution.

### AF Label Agreement with External Reference

To validate the reliability of our AF annotations in *MIMIC-III-Ext-PPG*, we compared them against an external reference dataset provided by Bashar *et al*.^14,15^, which is also derived from MIMIC-III, but provides AF annotations derived from cardiologists’ review of the ECG. Here, we use the AF episode annotations, which include patient identifiers and time stamps. We identified approximately 137 hours of overlap (16,476 × 30-second segments) between our dataset and the reference dataset by matching subject IDs and time intervals. Label agreement was evaluated only for those overlapping segments, allowing us to validate our annotations against expert-reviewed labels.

**Fig. 3:**
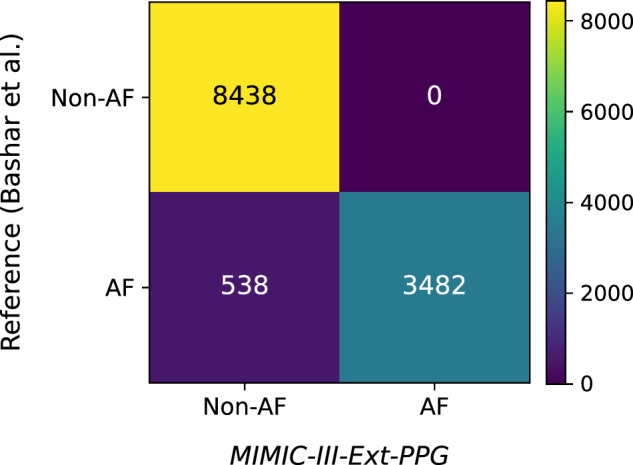
Confusion matrix showing segment-level agreement between *MIMIC-III-Ext-PPG* labels and the expert-annotated Bashar *et al*.’s reference.

Table 4 presents the distribution of the event rhythm labels in the segments of *MIMIC-III-Ext-PPG*’s which overlap with Bashar *et al*.’s annotations. Amongst those segments containing data labelled as AF by Bashar *et al*., 86.61% were also labeled as AF in *MIMIC-III-Ext-PPG*, with smaller proportions labeled as AFLT, 7.04%, SR, 6.00%, and SVATCH, 0.35%. It is worth stressing that signal annotations in Bashar *et al*.’s dataset often cover several hours and, therefore, might unintentionally include short intermittent SR and SVATCH episodes. To further quantify this agreement, segment-level validation metrics were computed, yielding a sensitivity of 0.86 (95% CI:0.85-0.87) and a specificity of 1.00 (95% CI: 0.99-1.00) for AF labeling (Fig. 3). All of the segments containing data labelled as non-AF by Bashar *et al*. were also labeled as non-AF in *MIMIC-III-Ext-PPG*, with most labeled as sinus rhythm (SR, 57.22%) or sinus tachycardia (STACH, 23.86%).

**Table 4:** Segment-level validation of *MIMIC-III-Ext-PPG* labels against overlapping expert-annotated Bashar *et al*.’s reference^14^.

Bashar *et al*.’s reference / Validation metric	Corresponding labels in *MIMIC-III-Ext-PPG* / Value
AF	**86.61% AF**, 7.04% AFLT, 6.00% SR, 0.35% SVTACH
Non-AF	57.22% SR, 23.86% STACH, 8.52% SBRAD, 6.34% 1AVB, 3.86% VPACE, 0.20% RBBB
Sensitivity (Recall)	0.86 (95% CI: 0.85–0.87)
Specificity	1.00 (95% CI: 1.00–1.00)
Precision	1.00
Accuracy	0.95

### Signal Quality and Physiological Parameter Distributions

**Fig. 4:**
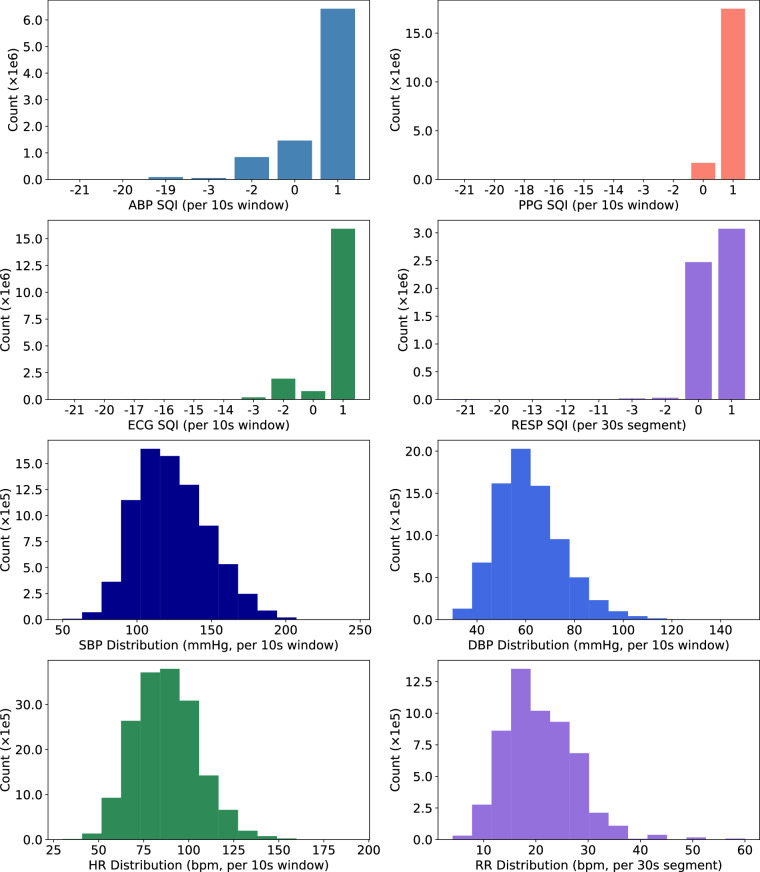
Overview of final signal quality and physiological parameter distributions. The top two rows show the distribution of SQIs for ABP, PPG, ECG (computed per 10-second window), and RESP (computed per 30-second segment) using rule-based algorithms. An SQI value of 1 indicates high quality, 0 indicates low quality, and negative values indicate that the SQI algorithm failed. The bottom two rows illustrate the distributions of SBP, DBP, HR, and RR extracted from the same signal segments.

Figure 4 presents a final overall view of signal quality and physiological parameter distributions in the dataset. The top two rows show the frequency of SQI values across ABP, PPG, ECG, and RESP signals. SQIs are calculated per 10-second window for ABP, PPG, and ECG, and per 30-second segment for RESP, using domain-specific rule-based methods. The bottom two rows present the distributions of SBP, DBP, HR, and RR, extracted from the same signal windows.

## Usage Notes

### Accessing the data

The waveforms are provided in WFDB format, ensuring compatibility with a wide range of signal processing tools such as PhysioNet’s WFDB software (which is available in Python, C, and MATLAB toolboxes), Python libraries (e.g., wfdb^16^, biosppy^17^, neurokit2^10^), as well as MATLAB packages^18^. The metadata is provided as a flat CSV file, which is also easy to process in any programming language.

### Possible use-cases

We envision that the dataset can be used for various purposes. Most notably, it can be used to train and validate PPG-based arrhythmia detection algorithms (e.g.,^19^). Here, different scenarios are conceivable, such as training robust detection algorithms for AF detection, considering the remaining heart rhythm types as non-AF, or building a comprehensive heart rhythm detector for PPG data. Secondary use cases include BP estimation (e.g.,^20^), HR estimation, or RR estimation, but also vascular age estimation (taking chronological age as a reference). The joint availability of all these labels will also allow for investigating multi-task prediction models or stratifying model performance in one prediction task according to one of the other categories.

The dataset is organized in a modular manner to support future extensions, such as the integration of additional waveform modalities and the improvement in annotations in accordance with the developments in signal processing techniques, as well as the application of the pipeline to other raw PPG datasets, e.g., MIMIC-IV waveform data once they become available. While *MIMIC-III-Ext-PPG* already provides rich clinical metadata, future work may further exploit additional clinical data available in MIMIC-III, such as vital signs, laboratory measurements, and additional clinical metadata, to enable multimodal analyses and more comprehensive modeling studies.

For comparability, we propose to use folds 0-6 for training, fold 7 for validation, and folds 8 and 9 for testing.

### Possible misuse or overinterpretation

It is also noteworthy to mention that the data is only for research purposes. It is not recommended to use the data as the basis of clinical decisions without further validation. In essence, the models trained from the data should not be regarded as validated clinical tools of diagnosis or monitoring. Similarly, the results of the models should not be regarded as generalizing beyond the clinical environment without further validation.

## Supplementary information

Supplementary Information

## Data Availability

The dataset is publicly accessible through the PhysioNet^1^ and contains physiological waveform data stored in WFDB format alongside corresponding patient metadata in CSV format. The organizational structure of the waveform files and detailed descriptions of all metadata columns are outlined in the Data Records section.

## Code availability

This work is exclusively based on publicly available datasets. The corresponding source code is available^21^.
