# Supplementary material for: MIMIC-III-Ext-PPG, a PPG-based Benchmark Dataset for Cardiovascular and Respiratory Signal Analysis
Source: Sci Data. 2026 Apr 28;13:668. doi: 10.1038/s41597-026-07335-8 (PMC13125301; doi:10.1038/s41597-026-07335-8)
Supplement: Supplementary file 1 — Supplementary Information [file 41597_2026_7335_MOESM1_ESM.pdf]

Supplementary Material for *MIMIC-III-Ext-PPG*, a PPG-based Benchmark Dataset for Cardiovascular and Respiratory Signal Analysis

Mohammad Moulaeifard[1], Marie Kutscher[1], Philip J. Aston[2,3], Peter H. Charlton[4], Nils Strodthoff[1*]

Table S1

Table S1: Overview of the SQI code map. Each row describes the SQI code assigned during preprocessing and validation for various physiological signals. The codes indicate whether the signal passed quality checks, failed specific criteria, or encountered structural issues such as missing data or insufficient length.

SQI Code	Stage / Source	Signal	Meaning
1	SQI Calculation	All	High-quality signal suitable for analysis
0	SQI Calculation	All	Low-quality signal, yet suitable for analysis
-2	Validation	All	Flatline signal or repeated extreme values detected
-3	Validation	All	Signal contains NaN values or is empty
-11	SQI Calculation	RESP	No valid peaks or troughs detected
-12	SQI Calculation	RESP	Insufficient number of valid respiratory cycles
-13	SQI Calculation	RESP	Insufficient valid excerpts for template matching
-14	SQI Calculation	ECG/PPG	No valid RR intervals detected (insufficient beats)
-15	SQI Calculation	ECG/PPG	RR interval too short for reliable evaluation
-16	SQI Calculation	ECG/PPG	Insufficient valid excerpts for template matching
-17	SQI Calculation	ECG	Invalid or insufficient R-peaks detected.
-18	SQI Calculation	PPG	Invalid or insufficient PPG peaks detected.
-19	SQI Calculation	ABP	ABP beat onset detection failed
-20	Dispatcher	All	Unknown signal type
-21	Exception handler	All	Unhandled exception during SQI computation

Table S2

Table S2: Dataset Variables Description.

Variable Name (type)	Description	Units / Allowed values
**Signal Information**		
record_id *(string)*	Identifier of the original MIMIC-III WFDB waveform record.	WFDB record ID (e.g., "3238451_0005")
event_id *(integer)*	Sequential index of the heart rhythm chart event within a record.	$0, 1, 2, \ldots$
segment_id *(integer)*	Index of the 30-second waveform segment counted backwards from the event time (segment 0 ends exactly at the event).	$0, 1, 2, \ldots$
signal_file_name *(string)*	Filename of the WFDB file storing the extracted 30-second segment.	<record_id>_<event_id>_ <segment_id>
patient *(string)*	Patient folder identifier used in the MIMIC-III waveform database.	Matches MIMIC-III Database
folder_path *(string)*	Relative path to the directory containing the segment files.	MIMIC-style directory
start_segment *(date/time)*	Start timestamp of the 30-second segment.	Matches MIMIC-III Database
start_record *(date/time)*	Start timestamp of the record from which the segments are extracted.	Matches MIMIC-III Database
event_time *(date/time)*	Timestamp of the underlying rhythm chart event.	Matches MIMIC-III Database

Table S2 – continued from previous page

Variable Name	Description	Units / Allowed values
event_rhythm *(string)*	Harmonized heart rhythm label associated with the chart event.	26 allowable codes: SR, STACH, SBRAD, AF, AFLT, APACE, VPACE, AVPACE, 1AVB, 2AVBM1, 2AVBM2, 3AVB, RBBB, LBBB, SARRH, JTACH, JR, SVTACH, MATACH, VTACH, VFIB, IDIOV, ASYS, WAPACE, OTHER, PATACH
**Subject Demographics**		
subject_id *(integer)*	Pseudonymized subject identifier. It is obtained from the patient identifier by dropping the leading "p" and converting the remaining digits to an integer value (e.g., p000052 $\rightarrow$52).	Unique per subject
hadm_id *(integer)*	Hospital admission identifier associated with the chart event.	Matches MIMIC-III Database
icustay_id *(integer)*	ICU stay identifier.	Matches MIMIC-III Database
clinical_information_system *(string)*	Clinical information system.	"Metavision", "Carevue"
age *(float)*	Patient age at hospital admission.	years / Matches MIMIC-III Database
weight *(float)*	Body weight.	kg / Matches MIMIC-III Database
height *(float)*	Body height.	cm / Matches MIMIC-III Database
gender *(string)*	Biological sex recorded in MIMIC-III.	M, F
ethnicity *(string)*	Harmonized ethnicity category.	White, Black, Hispanic, Asian, Other
**Blood Pressure, Heart Rate, and Respiratory Rate**		
vector_10s_median_sbp *(1D array (3,) of floats)*	Median SBP computed independently within each 10-second window.	mmHg

Table S2 – continued from previous page

Variable Name	Description	Units / Allowed values
vector_10s_iqr_sbp *(1D array (3,) of floats)*	Interquartile range of SBP computed within each 10-second window.	mmHg
vector_10s_median_dbp *(1D array (3,) of floats)*	Median DBP computed independently within each 10-second window.	mmHg
vector_10s_iqr_dbp *(1D array (3,) of floats)*	Interquartile range of DBP computed within each 10-second window.	mmHg
median_30s_sbp *(float)*	Median SBP across the full 30-second segment.	mmHg
iqr_30s_sbp *(float)*	Interquartile range of SBP across the full 30-second segment.	mmHg
median_30s_dbp *(float)*	Median DBP across the full 30-second segment.	mmHg
iqr_30s_dbp *(float)*	Interquartile range of DBP across the full 30-second segment.	mmHg
vector_10s_hr *(1D array (3,) of floats)*	HR calculated from ECG-derived RR intervals within each 10-second window.	bpm
median_30s_hr *(float)*	Median HR across the full 30-second segment.	bpm
iqr_30s_hr *(float)*	Interquartile range of HR across the full 30-second segment.	bpm
median_30s_rr *(float)*	Median RR derived from the RESP signal across the full 30-second segment.	breaths/min
iqr_30s_rr *(float)*	Interquartile range of RR across the full 30-second segment.	breaths/min
**Signal Quality and Morphological Features**		
vector_10s_pleth_sqi *(1D array (3,) of integers)*	PPG signal quality index, evaluated independently for each 10-second window (0–10 s, 10–20 s, 20–30 s).	{1, 0, –2, –3, –14, –15, –16, –18, –20, –21}

Table S2 – continued from previous page

Variable Name	Description	Units / Allowed values
vector_10s_ecg_sqi *(1D array (3,) of integers)*	ECG signal quality index, evaluated independently for each 10-second window.	{1, 0, –2, –3, –14, –15, –16, –17, –20, –21}
vector_10s_abp_sqi *(1D array (3,) of integers)*	ABP signal quality index, evaluated independently for each 10-second window.	{1, 0, –2, –3, –19, –20, –21}
resp_sqi *(integer)*	Signal quality index of the RESP waveform across the entire 30-second segment.	{1, 0, –2, –3, –11, –12, –13, –20, –21}
**Diagnostic Codes**		
icd9 *(string list)*	ICD-9 diagnosis codes associated with the hospital admission.	Standard ICD-9
icd10_truncated *(string list)*	Mapped and truncated ICD-10 diagnosis codes.	3-digit ICD-10
**Stratification**		
strat_fold *(integer)*	Stratified fold index for reproducible machine-learning benchmarking.	0–9

Figure S1

```
p00
  |-- p000052
  |   |-- 3238451_0005_0_1.hea
  |   |-- 3238451_0005_0_1.dat
  |   |-- ...
  |-- p000107
  |   |-- 3805787_0011_0_4.hea
  |   |-- 3805787_0011_0_4.dat
  |   |-- ...
...
p09
  |-- p090012
  |   |-- 3522957_0007_0_0.hea
  |   |-- 3522957_0007_0_0.dat
  |   |-- ...
```

Figure S1: Folder structure of the dataset uploaded to PhysioNet.
